# Heavy consumption of dental services; a longitudinal cohort study among Finnish adults

**DOI:** 10.1186/1472-6831-13-18

**Published:** 2013-04-24

**Authors:** Annamari Nihtilä, Eeva Widström, Outi Elonheimo

**Affiliations:** 1 Helsinki Health Centre and Institute of Dentistry, University of Helsinki, Helsinki, Finland; 2 Network of Academic Health Centres, Institute of Clinical Medicine, Department of General Practice and Primary Health Care, University of Helsinki, Helsinki, Finland; 3 National Institute for Health and Welfare, Helsinki, Finland; 4 Institute of Clinical Dentistry, University of Tromso, Tromso, Norway

**Keywords:** Health services research, Dental services, Longitudinal register study, Public dental service, Adult and elderly population, Complicated treatment needs, Heavy users of oral health services

## Abstract

**Background:**

A reform to Dental Care legislation in 2002 abolished age limits restricting adults’ use of public dental services in Finland. In the Public Dental Service (PDS) unit of Espoo, the proportion of adult patients rose from 36% to 57%. The aim of this study was to investigate heavy use of dental services by adults and its determinants.

**Methods:**

A longitudinal cohort study was undertaken based on a PDS patient register. Of all adults who attended the PDS in Espoo in 2004, those who had six or more visits (n=3,173) were assigned to the heavy user group and a comparison group of low users (n=22,820) had three or fewer dental visits. A sample of 320 patients was randomly selected from each group. Baseline information (year 2004) on age, sex, number and type of visit, oral health status and treatment provided was collected from treatment records. Each group was followed-up for five years and information on the number and types of visit was recorded for each year from 2005 to 2009.

**Results:**

Most heavy users (61.6%) became low users and only 11.2% remained chronic heavy users. Most low users (91.0%) remained low users. For heavy users, the mean number of dental visits per year (3.0) during the follow-up period was significantly lower than initially in 2004 (8.3) (p<0.001) but 74.8% of heavy users had had emergency visits compared with 21.6% of the low users (p<0.001).

A third (33%) of the visitors in each group had no proper examination and treatment planning during the 5-year follow-up period and two or more examinations were provided to fewer than half of the heavy (46.1%) or low (46.5%) users.

The mean number of treating dentists was 5.7 for heavy users and 3.8 for low users (p<0.001).

**Conclusions:**

Frequent emergency visits were characteristic of heavy users of dental services. Treatment planning was inadequate, probably partly due to the many dentists involved and too many patients requesting care. Better local management and continuous education are needed to ensure good quality adult dental care and to reduce heavy consumption.

## Background

Heavy use of dental services is a major drain on resources, but the reasons and patterns of heavy use of dental services have been little investigated. There is no consensus definition for heavy use of dental services. In primary medical health care, the 10% of persons making the most visits have most often been defined as frequent attenders [1]. From previous studies in primary medical health care settings, we know that frequent attendance may or may not be persistent [2]. In a study in the UK, approximately 30% of heavy consumers remained frequent attenders the next year [3], and, according to a Swedish study, 14% of the frequent attenders persisted after five years [4]. We found no longitudinal studies assessing persistent heavy use of dental services.

A number of factors influence the use of dental care. A theoretical model by Andersen and Newman stresses the importance of characteristics of the oral health service delivery system, changes in medical technology and social norms relating to the definition and treatment of illness, and individual determinants of utilization [5]. Many studies have confirmed the independent relationships between patterns of dental care utilization and individual factors, such as socio-demographic factors [6-11] perceived quality of dental care [12], type of dental care utilized [13] and self-reported oral problems [7,11]. Most of the studies dealing with oral health services utilization have focused on the individual characteristics while less attention has been paid to societal determinants and health service delivery systems, although they frame the provision of services to the individual [5].

The oral health care provision system in Finland is consistent with the Nordic model typical for the Scandinavian countries [14]. In this model, a Public Dental Service (PDS) employing salaried personnel, run by county councils or municipalities and financed mainly by tax revenues, is responsible for organizing dental care for certain population groups, e.g. children and adolescents and some groups of the elderly or, in some countries, for all those who wish to use the service. Care in the PDS is free for children and youngsters and in some countries also for certain groups of adults. In general, treatment of adults is subsidized and fixed fees are used. In all Nordic countries there is also a private sector, which part of the population (usually those with higher income and education) chooses to use. Private treatment may also be subsidized through national insurance systems [15].

In Finland, between 1956 and 1980, the PDS catered mainly for children and youngsters and adults were supposed to visit private dentists or denturists. In the 1980s, young adults were successively given access to the PDS, age group by age group. Some special needs groups and World War II veterans were included in the 1990s.

In 2001, when the age limit for access to the PDS was ‘born in 1956 or later’ the dental care provision system was reformed and the age limits restricting adults' use of the PDS were abolished. At the same time, all adults who used the private sector, irrespective of age, became entitled to partial reimbursement of the cost of care from the National Health Insurance [16].

The Dental Care Reform aimed to increase equity by improving adults’ access to care and reducing cost barriers. A premise of this Reform was that oral health care should be distributed primarily according to dental needs [16] and no longer according to age group or having been a patient earlier. The magnitude of the Reform can be seen in the fact that about 40% of Finnish adults in a short period became eligible to use the PDS. This resulted in long waiting lists to the PDS, especially in the bigger cities, partly because treatment in the PDS was cheaper than in the private sector, even after the reimbursement of private care [16]. In 2005, Care Guarantee legislation was introduced in health care including public dental care. This stated that emergency services and non-urgent treatments had to be provided within clear time frames. In the PDS, this meant that emergency services should be given immediately or within three days and non-urgent care within six months to all those who requested and needed it.

Espoo, close to the capital, Helsinki, is the second largest city in Finland. Despite a good supply of private dental services for adults in the capital region, the Dental Care Reform put pressure on the PDS in Espoo. Before the Dental Care Reform Act, until 2001, the PDS of Espoo treated mainly children and young adults up to the age of 30 years and small numbers of older special needs patients. As a result of the reform, adults made up a greater proportion of patients in the PDS of Espoo (36.2% in 2000 and 56.9% in 2009). The PDS operates 27 clinics and patients are free to choose where to go.

In order to make the PDS more effective in Espoo, two studies were conducted to identify heavy users and reasons for heavy use of dental services [17,18]. These studies showed that 7.0% of the children and youngsters and 10.5% of the adults who had visited the PDS in Espoo were heavy users in 2004. Their visits accounted for 26.3% of all visits by children and youngsters and 31.6% of all adult dental visits. Need for complicated treatment, lack of experience of adult dental care among dentists and dental hygienists and lack of specialists in the PDS resulted in high numbers of dental visits for a number of adult patients [17]. For children, our study revealed two main reasons for heavy use: high amounts of orthodontic treatment provided by general dentists and high numbers of decayed teeth in a small number of children [18].

Our primary objective was to investigate whether or not the adult heavy users persisted as heavy users during the five years following the baseline year. The second objective was to analyse whether the treatment provided differed between baseline heavy and low users of dental services during the follow-up period. We also wanted to study determinants of persistent heavy use of dental services.

## Methods

**Figure 1:**
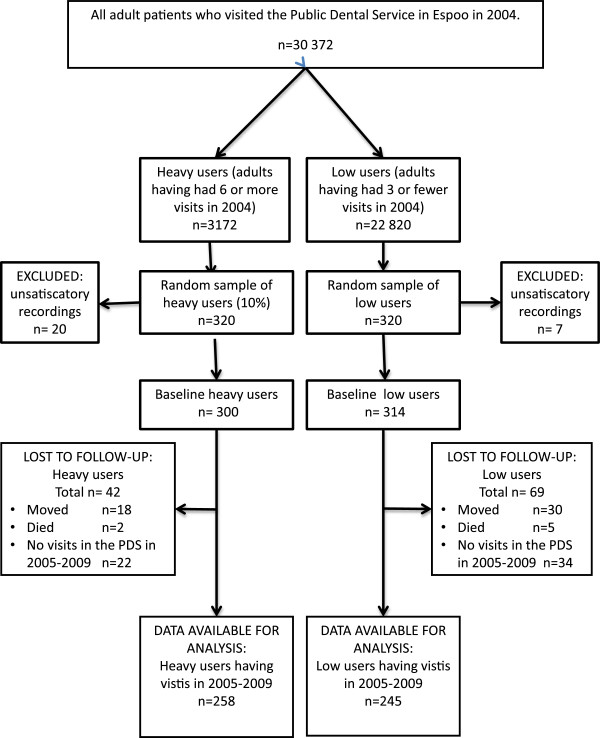
Selection of the study participants from the patient register in the PDS of Espoo.

We used a longitudinal cohort study design and followed the heavy and low users during a five-year period. All adults who had made six or more visits to dentists or dental hygienists in the PDS in 2004 were initially defined as heavy consumers of dental services. They accounted for the top 10.5% of all adult visits. Low consumers were those who had had three or fewer visits during that year. There were 3,172 heavy user patients and 22,820 low user patients by these criteria in the patient register of the PDS in Espoo that year. A sample of 320 patients (10% of the heavy users and a comparison group of equal size of the low users) was randomly drawn from each group (Figure 1). The city administration of Espoo, the legal owner of the patient register, granted research permission. In the next step, information on age, sex, occupation and self-reported general health status was collected from the patient records. From these records we also collected information on the number of visits and on all treatment measures the patients were given during the visits.

Patients’ occupational status was categorized into six classes: upper-level white-collar workers, lower-level white-collar workers, blue-collar workers, students, pensioners and others (housewives, unemployed), using the classification recommended by Statistics Finland [19]. All the information except treatment measures had to be collected separately by hand for each patient from the patient records. This was done by one of the authors (AN).

Accurate information on the dental visits was found for 300 heavy and 314 low users from the patient records out of the sample of 320 in 2004. In the follow-up study, we included all the heavy and low consumers of dental services identified in 2004 who had visited the PDS in Espoo during 2005–2009 (Figure 1). The difference in the mean age between the heavy (36.9 years) users and low users (39.2 years) with no visits was not statistically significant.

Numbers and types of visits, and treatment provided according to the Finnish Social Insurance Institution classification were collected for each year as well as the number of treating dentists.

Data were analysed by means of SPSS version 18 (Statistical Package for the Social Sciences). Differences between the heavy and low consumers of dental services were evaluated by Chi-square and Mann–Whitney tests. Differences between the baseline and follow-up groups were evaluated by Chi-square and Wilcoxon tests. Predictors of chronic heavy consumption were analysed by logistic regression analysis.

## Results

### Service utilization patterns during follow-up

As can be seen in Figure 1, a slightly greater proportion of initial heavy users (86.0%) revisited the PDS of Espoo during 2005–2009 compared with low users (78.0%) (p<0.05). A small number (29; 11.2%) of the initial heavy users persisted as heavy users through the follow-up period and had six or more visits each year (according to our heavy user criteria). Of the initial low users, five persons (2%) became heavy users. Most of the low users (91.0%) remained low users (had less than 16 visits during 2005–2009) and 61.6% of the heavy users changed to the low user category. The rest of the heavy (17.2%) and low users (7.0%) became “intermediate users” who had had 16–29 visits.

For heavy users, the mean number of all dental visits per year during the follow-up period (3.0) was significantly lower than initially in 2004 (8.2). The mean number of visits remained twice as great compared with the low users (Table 1). In the low user group, no significant differences could be seen. About half of the heavy users (53.5%) and 49.0% of the low users (p=ns) had visited a dental hygienist during the follow-up period. The use of hygienist services had increased slightly.

**Table 1:** Service utilisation characteristics of the heavy and low users of the PDS of Espoo in baseline year 2004 and during the follow-up period 2005–2009

	**Heavy users**			**Low users**		
	**Baseline n=300**	**Follow-up n=258**		**Baseline n=314**	**Follow-up n=245**	
	**Year 2004**	**Per year 2005-2009**	**Total 2005-2009**	**Year 2004**	**Per year 2005-2009**	**Total 2005-2009**
Mean number of visits including examinations	0.5	0.2	1.1	0.4	0.2	1.0
Mean number of emergency visits	1.3	0.6	2.9	0.6	0.06	0.3
Mean number of other visits to dentist	5.5	1.8	9.0	0.3	1.04	5.2
Mean number of visits to a dentist	7.3	2.6	13.0	1.3	1.3	6.5
Mean number of visits to a dental hygienist	0.9	0.4	2.0	0.4	0.3	1.2
Mean number of all dental visits	8.2	3.0	15.0	1.7	1.6	7.7

p<0.001¸ p<0.05.

### Characteristics of heavy and low users

The proportion of men was greater in the heavy than in the low users group. Heavy users were older, to greater extent pensioners, and had lower social status than the low users (Table 2). The chronic heavy users were even older, 82.4% of them were 45 years or older. Heavy users also reported more often general illnesses (39.9%) than the low users (23.8%) (p<0.001).

**Table 2:** Demographic characteristics of the baseline and follow-up heavy and low users of the PDS of Espoo in 2004

	**Heavy users**		**Low users**	
	**Baseline n=300**	**Follow-up 2005–2009 n=258**	**Baseline n=314**	**Follow-up 2005–2009 n=245**
Sex: Women% in 2004	55.0	55.8	65.0	63.5
Men % in 2004	45.0	44.2	35.0	36.5
Mean age in 2004 (years)	47.9	49.7	41.4	42.0
18–29-years% in 2004	19.7	16.7	23.6	19.7
30–44-years % in 2004	28.0	26.4	45.5	48.0
45–64-years % in 2004	31.7	34.1	19.8	21.7
65+ years % in 2004	20.6	22.9	11.1	10.7
Upper-level white-collar workers % in 2004	9.4	9.0	18.6	18.4
Lower-level white-collar workers % in 2004	25.7	27.1	33.2	35.7
Blue-collar workers % in 2004	27.7	26.0	16.9	18.4
Students% in 2004	9.3	7.4	12.8	8.6
Pensioners % in 2004	20.3	22.9	10.5	9.4
Others% in 2004	7.7	7.8	8.0	9.4

### Treatments provided

**Figure 2:**
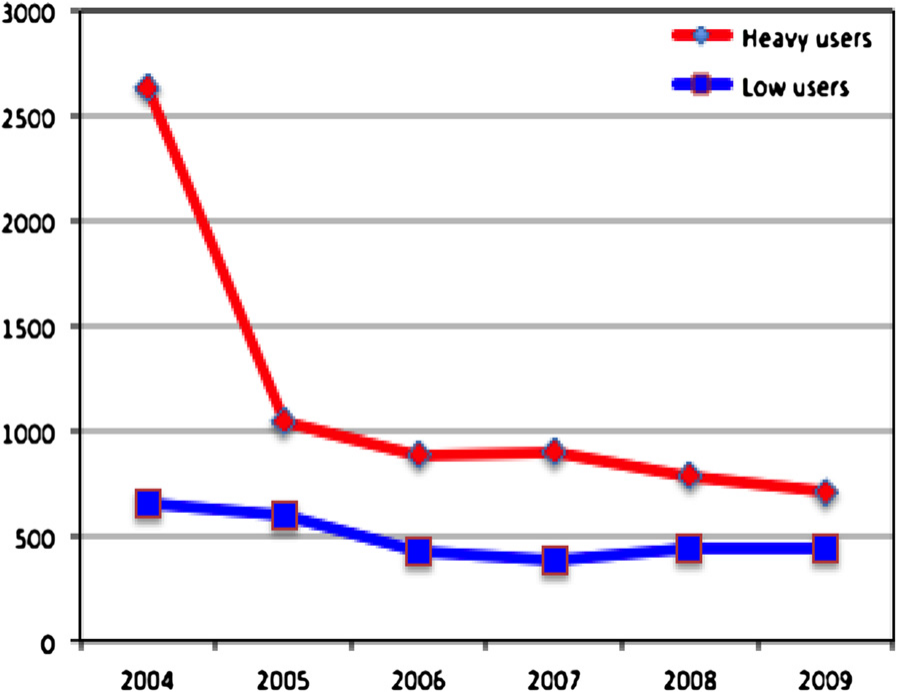
Total numbers of treatment procedures for heavy and low users during 2004–2009.

The total number of treatment measures decreased by 60.4% one year after baseline and the decrease was 73.2% after five years for heavy users. For low users, the total number of treatment measures decreased after one year by 8.3% and after five years the decrease was 31.7% compared with the baseline (Figure 2). During the follow-up period, heavy users had a significantly higher number of treatment measures except for examinations and treatment planning, compared with the low users. Examinations were not common; on average, only one examination per patient had been provided during the five-year period (Table 1). There were no significant differences between the proportion of heavy users (67.0%) and low users (66.9%) whose complete oral health status was recorded during the follow-up period. Two or more examinations during 2004–2009 were provided to 46.1% of the heavy and 46.5% of the low users.

Emergency visits were common for heavy users (Table 1); 74.8% had such visits during 2005–2009 compared with 21.6% of the low users (p<0.001). The proportion of low users who had emergency visits decreased significantly. The highest number of emergency visits per person during the 5-year follow-up period was 24 in the heavy user group and six in the low user group. Almost all (96.6%) chronic heavy users had made emergency visits; their mean number of emergency visits per year was 1.5. Need for an emergency appointment was assessed by dental assistants on phone. Specific criteria were used in this assessment: pain, bleeding, trauma etc. but also broken restorations.

### Numbers of treating dentists

The number of dentists seen by the patients during the study period 2004–2009 ranged from 1–21 (the mean being 5.7) for heavy users and from 0–23 (the mean being 3.8 for low users (p<0.001). For persons who had made 30 visits or more, the mean number of dentists seen was 9.4.

### Reasons for chronic heavy use of dental services

We used logistic regression analysis to investigate whether any variables were statistically significantly associated with chronic heavy use of dental services (Table 3). We found that, compared with the persistent low users, the chronic heavy users had higher odds of being 65 years or older, having the social status of pensioners (OR=10.0, p< 0.05) and having had emergency treatments (OR=7.9, p < 0.001). When adjusted for gender and age, having had five or more different treating dentists remained a significant risk variable (OR=14.4, p < 0.001).

**Table 3:** Predictors of persistent heavy use of oral health services

	**N=252**	
**Independent variables**	**OR (95% CI)**	**p-value**
**Sex**		
Female	1.0 (0.4, 2.1)	0.917
Male	Reference	
**Age group**		
30-44	0.4 (0.1, 2.2)	0.307
45-64	4.4 (1.2, 16.6)	0.027
65+	6.7 (1.7, 26.6)	0.007
18-29	Reference	
**Occupational status**		
White-collar workers	1.6 (0.2, 12.9)	0.685
Blue-collar workers	3.7 (0.4, 31.8)	0.230
Pensioners	10.0 (1.2, 85.6)	0.036
Students	Reference	
**General health status**		
Self-reported chronic illnesses	1.6 (0.7, 3.9)	0.276
No chronic illnesses	Reference	
**Number of treating dentists**		
5 or more	14.4 (5.5, 38.0)	0.000
1-4	Reference	
**Type of treatment measures**		
Emergency	7.9 (3.0, 20.7)	0.000
No emergency treatments	Reference	
Examination	2.6 (1.7, 4.2)	0.000
No examinations	Reference	

Persistent heavy users (n=29) are compared with persistent low users (n=223). Odds ratios and 95% confidence limits based on logistic regression.

## Discussion

Espoo is a wealthy area where employment rate, income and education levels are well above national average, with rather stable population of 252 000 inhabitants. Thus a great majority of the initial study subjects (81.9%) could be followed during a longer time period. In addition, half of the drop-outs could be confirmed no longer to be living in Espoo and thus not have access to the PDS. A small proportion (9.1%) stayed in Espoo but did not visit the PDS after 2004. They were probably private dentist patients using the PDS occasionally or became private dentist patients after seeing the long waiting time for the PDS.

A limitation of our study is the lack of information concerning the possible dental visits to the private sector, but it has traditionally not been common to use both sectors in Finland. Private dentists regularly recall most of their patients [20] and a recent questionnaire study of middle aged Finnish adults living in the Helsinki metropolitan region in 2007 showed that slightly fewer than half of them would have been able to pay for private care. The same study also showed that only 9.2% of the respondents had used both sectors [21]. Our results are also limited to one PDS unit and thus cannot be extrapolated to all the PDS units in Finland.

In all municipal PDS units in Finland, dentists are obliged to use standardized dental records and treatment item codes defined by the Finnish Social Insurance Institution. These codes, based on examinations and treatments provided, are used to pay dentists productivity bonus fees in addition to their monthly salaries. This encourages careful recording and therefore we think that our data are reasonably valid and reliable.

The study showed that, after the baseline year 2004, the mean number of visits made by the heavy users declined considerably and was only slightly higher than the mean number of visits made by adults in general in the PDS of Espoo. This varied from 2.7 to 2.9 visits per year during 2005–2009. A number of the initial high users were probably “new patients” in 2004 who had qualified for access to the PDS after the end of 2002, when the age limits restricting adults’ use of the PDS were abolished. The high cost of private dental care may have created an accumulated treatment need in this group. The chronic heavy users group was older and included more retired persons (income usually decreases on retirement) than the other baseline heavy users. The positive finding from our study, that only a small proportion of the heavy users (11.2%) remained chronic heavy users, supports this interpretation

In spite of high numbers of visits and treatments provided, especially in the heavy user group, during the follow-up period, the number of examinations, including treatment planning, was low. In the PDS, no universal recommendation exists for regular annual check-ups; individual recall intervals, often exceeding one year, are proposed for adult patients. Furthermore, after the Dental Care Reform, most PDS units were unable to recall any adult patients [22]. A third (33%) of persons in each group had no proper examinations during the follow-up period and two or more examinations were provided to fewer than half of the heavy (46.1%) or low (46.5%) users. Because examination is one of the treatment measures that gives the dentists good additional remuneration, it is unlikely that examinations would not have been recorded if they had been provided. It is more likely that many visits were emergencies or semi-emergencies, e.g. broken and lost fillings and endodontic treatments that were initiated without full examination. Our findings also suggested that chronic heavy use was related to high numbers of emergency visits.

Another obstacle to avoiding examinations has probably been the scarcity of personnel resources in the PDS of Espoo, especially directly after the dental care reform. High turnover of dentists and therefore lack of a stable dentist-patient relationship could also result in avoiding examinations and pushing patients towards rapid emergency treatment measures. This does not give a very good picture of the quality of care provided.

The frequent changes of treating dentists can also have had an impact on the treatment decision. It is well-known that treatment decision-making among dentists shows wide variation [23,24]. Changing dentist has also been reported to result in more restorative treatment [25]. The high numbers of different dentists involved, especially in the care of the chronic heavy users, may partly explain the elevated numbers of visits and treatment measures.

The dental treatment of heavy users and especially the persistent heavy users of care is demanding. Better access to dental specialists or medical doctors when needed would probably have helped in planning more comprehensive treatments. All patients and especially the persistent chronic heavy users should have been offered a team of responsible dentists and dental hygienists who could have shared the work. In all Nordic countries, dental hygienists are numerous and well educated and, especially in Finland, they could be used more in adult dental care [26].

Our study shows that when radical changes in the care provision system are implemented, like the Finnish Dental Care Reform, the care providers should receive the necessary resources, further education should be organized for the staff and clinical treatment routines should be adjusted accordingly. This requires determined leadership in the local level. Being a lead dentist in the PDS has not been a very attractive job in comparison with clinical work [27]. To be a leader was hard work after the Dental Care Reform, especially as half of the PDS dentists did not like the abolition of patient age limits, because this made their clinical work more difficult and introduced more emergency treatment [22].The PDS has an important new role in improving equity in the use of dental services in Finland, by supplying care to all adults who do not have the means for or do not wish to use the private sector. In publicly funded systems, efficient care production and cost-containment are important. The PDS should aim for good quality and cost-efficient treatment by offering comprehensive care and by avoiding unnecessary procedures.

## Conclusion

The study shows that a small proportion of initial heavy-users persisted as heavy users during a five-year study period, but most heavy users needed more treatment than the initial low users, whose treatment needs remained low. requent emergency treatments were characteristic of heavy users of services. Innovative changes in the organization of the PDS system and better local management are needed to ensure good quality adult dental care and to reduce heavy consumption of dental services.

## Competing interests

The authors declare that they have no competing interests.

## Authors’ contributions

AN participated in the design of the study, collected the data, performed the statistical analyses and drafted the manuscript. EW tutored, participated in the design of the study and drafted the manuscript. OE tutored and helped draft the manuscript. All authors have read and approved the final manuscript.

## Pre-publication history

The pre-publication history for this paper can be accessed here:

http://www.biomedcentral.com/1472-6831/13/18/prepub
